# Viral RNA level, serum antibody responses, and transmission risk in recovered COVID-19 patients with recurrent positive SARS-CoV-2 RNA test results: a population-based observational cohort study

**DOI:** 10.1080/22221751.2020.1837018

**Published:** 2020-11-05

**Authors:** Chao Yang, Min Jiang, Xiaohui Wang, Xiujuan Tang, Shisong Fang, Hao Li, Le Zuo, Yixiang Jiang, Yifan Zhong, Qiongcheng Chen, Chenli Zheng, Lei Wang, Shuang Wu, Weihua Wu, Hui Liu, Jing Yuan, Xuejiao Liao, Zhen Zhang, Xiaolu Shi, Yijie Geng, Huan Zhang, Huanying Zheng, Min Wan, Linying Lu, Xiaohu Ren, Yujun Cui, Xuan Zou, Tiejian Feng, Junjie Xia, Ruifu Yang, Yingxia Liu, Shujiang Mei, Baisheng Li, Zhengrong Yang, Qinghua Hu

**Affiliations:** a Shenzhen Center for Disease Control and Prevention, Shenzhen, People’s Republic of China; b State Key Discipline of Infectious Disease, National Clinical Research Center for infectious disease, Shenzhen Third People's Hospital, Second Hospital Affiliated to Southern University of Science and Technology, Shenzhen, People’s Republic of China; c Guangdong Provincial Center for Disease Control and Prevention, Guangdong, People’s Republic of China; d Shenzhen LongHua District Maternity and Child Healthcare Hospital, Shenzhen, People’s Republic of China; e State Key Laboratory of Pathogen and Biosecurity, Beijing Institute of Microbiology and Epidemiology, Beijing, People’s Republic of China

**Keywords:** COVID-19, SARS-CoV-2, recurrent positive, viral RNA level, antibody responses, transmission risk

## Abstract

Managing recovered COVID-19 patients with recurrent-positive SARS-CoV-2 RNA test results is challenging. We performed a population-based observational study to characterize the viral RNA level and serum antibody responses in recurrent-positive patients and evaluate their viral transmission risk. Of 479 recovered COVID-19 patients, 93 (19%) recurrent-positive patients were identified, characterized by younger age, with a median discharge-to-recurrent-positive length of 8 days. After readmission, recurrent-positive patients exhibited mild (28%) or absent (72%) symptoms, with no disease progression. The viral RNA level in recurrent-positive patients ranged from 1.8 to 5.7 log10 copies/mL (median: 3.2), which was significantly lower than the corresponding values at disease onset. There are generally no significant differences in antibody levels between recurrent-positive and non-recurrent-positive patients, or in recurrent-positive patients over time (before, during, or after recurrent-positive detection). Virus isolation of nine representative specimens returned negative results. Whole genome sequencing of six specimens yielded only genomic fragments. 96 close contacts and 1,200 candidate contacts of 23 recurrent-positive patients showed no clinical symptoms; their viral RNA (1,296/1,296) and antibody (20/20) tests were negative. After full recovery (no longer/never recurrent-positive), 60% (98/162) patients had neutralizing antibody titers of ≥1:32. Our findings suggested that an intermittent, non-stable excretion of low-level viral RNA may result in recurrent-positive occurrence, rather than re-infection. Recurrent-positive patients pose a low transmission risk, a relatively relaxed management of recovered COVID-19 patients is recommended.

## Introduction

Coronavirus disease 2019 (COVID-19), caused by severe acute respiratory syndrome coronavirus 2 (SARS-CoV-2), has spread globally to over 213 countries [1,2]. As of August 10, 2020, there have been more than 20,000,000 confirmed patients and 730,000 deaths. Currently, there are approximately 200,000 new confirmed patients daily, posing huge challenges for public health and medical institutions.

Worldwide, there are more than 12,000,000 recovered COVID-19 patients [2]. Recent reports have described recovered COVID-19 patients with recurrent positive reverse transcription quantitative PCR (RT-qPCR) test results for SARS-CoV-2 (recurrent-positive patients) [3–6]. These studies focused on the clinical characteristics of a small number (<40) of recurrent-positive patients and found that they generally showed no clinical symptoms or disease progression. However, their positive SARS-CoV-2 RNA test results suggest that these patients might be virus carriers. The management of recurrent-positive patients is challenging because of the current lack of understanding regarding their viral RNA level, antibody responses, and viral transmission risk. In China, recurrent-positive patients are placed under costly fourteen-day quarantine. Clarifying the characteristics and viral transmission risk of recurrent-positive patients is critical for appropriately managing these cases.

We performed a population-based observational cohort study of 479 recovered COVID-19 patients, discharged from February 1 to May 5, 2020 in Shenzhen, China. Based on the results of integrating RT-qPCR, antibody assays, neutralization assays, virus isolation, whole genome sequencing (WGS), and epidemiological investigation of close contacts, we comprehensively detailed the demographic, clinical, viral RNA level and antibody response characteristics and evaluated the viral transmission risk of recurrent-positive patients.

## Material and methods

### Patients

**Figure 1.**
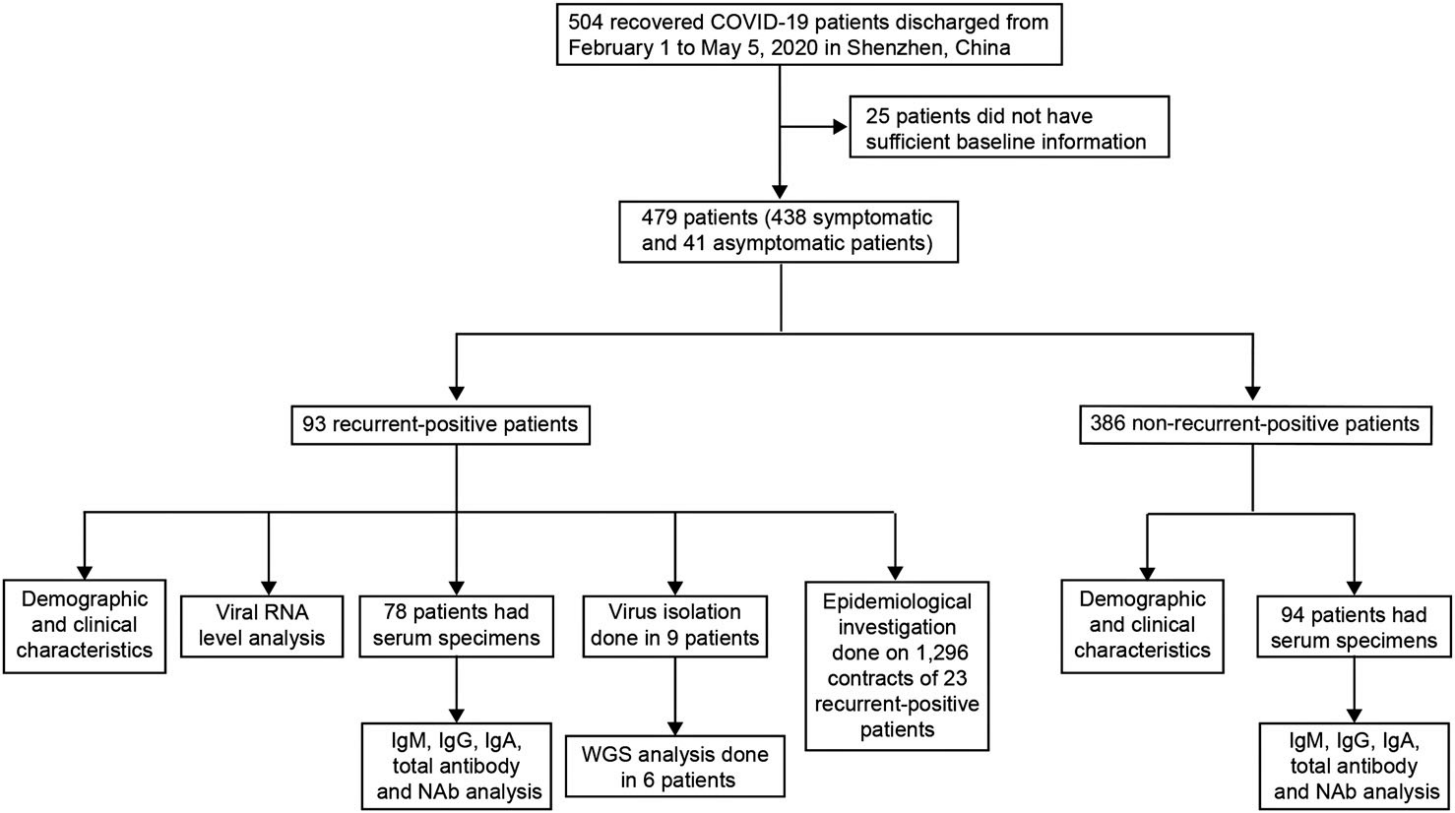
Profile of the recovered COVID-19 patients included in this study.

All COVID-19 patients in Shenzhen were treated at the designated Shenzhen Third People’s Hospital; their cases were reported to Shenzhen Center for Disease Control and Prevention (CDC) [7]. This study enrolled all recurrent-positive patients discharged from February 1 to May 5, 2020 in Shenzhen, including asymptomatic patients identified during the RT-qPCR screening of confirmed COVID-19 patient close contacts (Figure 1). Discharge criteria included: (1) normal temperature for >3 days, (2) resolved respiratory symptoms, (3) substantial pulmonary lesion absorption on chest computed tomography (CT) images, and (4) negative results from two consecutive SARS-CoV-2 RNA tests conducted >1 day apart. After discharge, recovered patients were quarantined at home (before February 18) or in centralized facilities (from February 18) for 14 days. During the 14-day quarantine period, both nasopharyngeal and anal swabs (*n* = 2,442, 4–20 per person) were collected from each patient on the 7th and 14th days (before March 18) or the 1st, 3rd, 7th, and 14th days (from March 18) for SARS-CoV-2 RNA detection by RT-qPCR. From March 18, serum specimens were collected on the 1st, 3rd, 7th, and 14th days for antibody assays (*n* = 499, 2–8 per person), and some recurrent-positive patient blood specimens (*n* = 147, 1–4 per person) were collected for SARS-CoV-2 RNA detection by RT-qPCR. After quarantine, recovered patients were regularly followed-up on the 7th, 14th, 30th, and 60th days post-discharge from hospital. Demographic and clinical severity information was extracted from electronic hospital medical records. Clinical severity on first admission was classified as asymptomatic, mild, moderate, or critical based on Chinese Guidelines for Diagnosis and Treatment for Novel Coronavirus Pneumonia [8].

The study was approved by the Ethics Committee of Shenzhen CDC (QS2020060007). As data collection is part of the public health investigation of an emerging outbreak, individual informed consent was waived.

### Case definition

**Figure 2.**
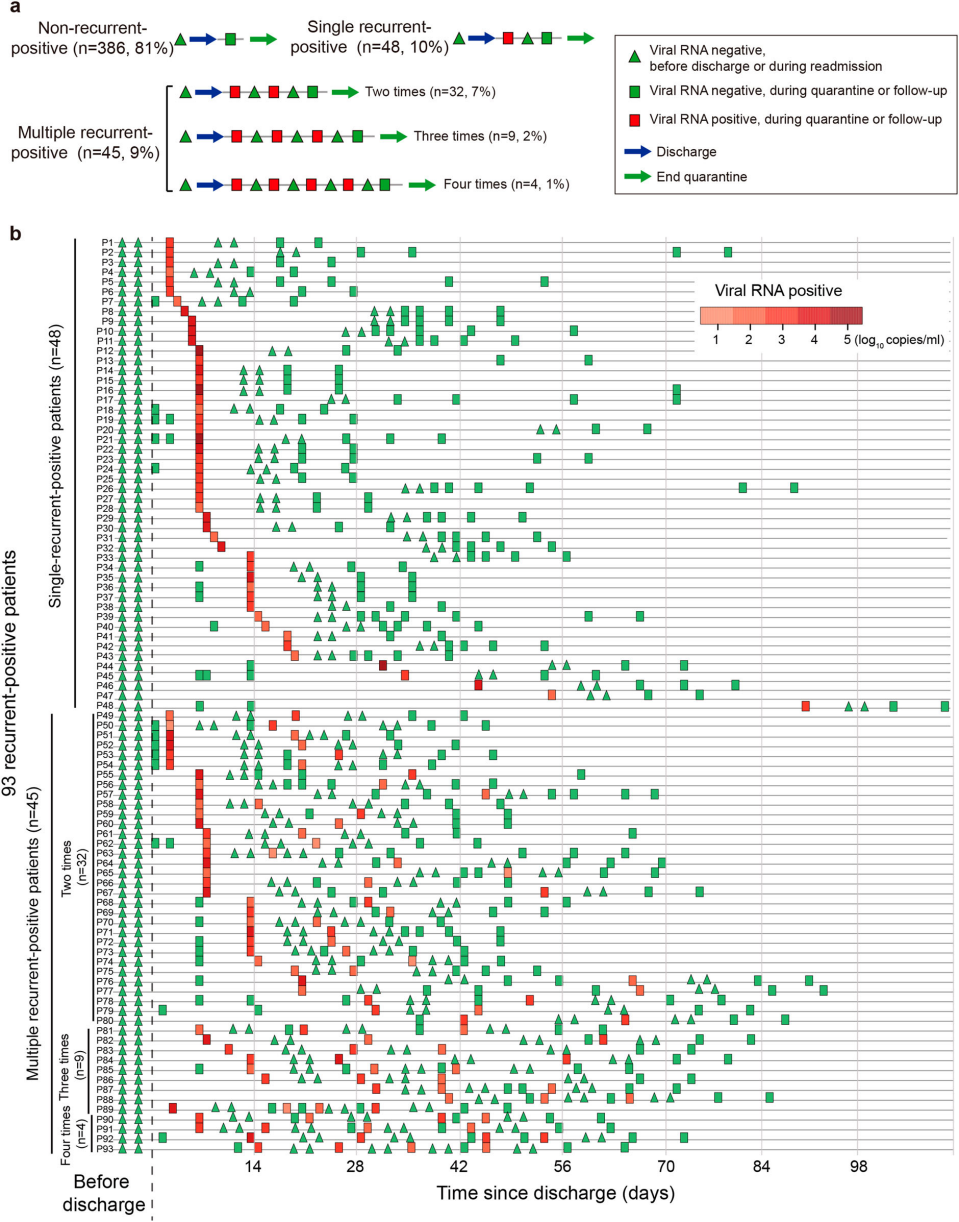
Recurrent-positive patient definition concept figure (a) and temporal distribution of the SARS-CoV-2 viral RNA level in 93 recurrent-positive patients (b). Red and green colours show viral RNA-positive and -negative tests, respectively. The triangles show viral RNA results from testing performed before discharge or during readmission. The rectangles show viral RNA results from testing performed during quarantine or follow-up.

Because negative results from two consecutive SARS-CoV-2 RNA tests were part of the discharge criteria, a recovered patient with recurrent-positive test results was defined as a recurrent-positive patient (Figure 2). These patients were readmitted to hospital for further medical observation until they met the discharge criteria again, including negative results from two consecutive SARS-CoV-2 RNA tests. After re-discharge, a recurrent-positive patient with further positive SARS-CoV-2 RNA test results was defined as a multiple-recurrent-positive patient. A recovered patient with constant negative SARS-CoV-2 RNA test results was defined as a non-recurrent-positive patient.

### Procedures

SARS-CoV-2 RT-qPCR tests were performed on the day of sampling using commercial kits (Zhongshan Daan Biotech). After 45 cycles, specimens with cycle threshold (Ct) values of ≤40 for both tested genes were considered positive; single-gene-positive specimens were retested and considered positive if the Ct values from the repeat tests were ≤40. The viral RNA level (copies/mL) was calculated from Ct values based on the standard curve of control product (Zhongshan Daan Bio-Tech, Figure S1). Serum immunoglobulin (Ig) antibody against the SARS-CoV-2 surface spike protein receptor-binding domain (RBD) was measured using a chemiluminescence kit (IgM, IgG, and total antibody, Beijing Wantai Biotech, measured by cut-off index [COI]) or enzyme-linked immunosorbent assay kit (IgA, Beijing Hotgen Biotech, measured by optical density at 450/630 nm [OD450/630]). The cut-off for seropositivity was set according to the manufacturer’s instruction, verified using positive (169 serum specimens from confirmed COVID-19 patients) and negative (128 serum specimens from healthy persons) controls, and both of sensitivity and specificity were 100%. Specimens with COI>1 (IgM, IgG, or total antibody), OD450/630 > 0.3 (IgA) were considered positive. Virus neutralization assays were performed using SARS-CoV-2 virus strain 20SF014/vero-E6/3 (GISAID accession number EPI_ISL_403934) in biosafety level 3 (BSL-3) laboratories. Neutralizing antibody (NAb) titer was the highest dilution with 50% inhibition of cytopathic effect, and a NAb titer of ≥1:4 was considered positive. Vero-E6 cells were used for virus isolation in a BSL-3 laboratory. WGS was performed after specifically amplifying SARS-CoV-2 RNA. Epidemiological investigations were conducted on 96 close contacts (unprotected exposure) and 1,200 candidate contacts of 23 recurrent-positive patients, identified during follow-up. Detailed methods are provided in the supplementary text.

### Statistical analysis

We performed statistical analyses using R version 3.6.1. Categorical and continuous variables were compared using Chi-square and Mann–Whitney U tests, respectively. Correlations were assessed using Spearman’s correlation test. For all tests, *p *< 0.05 was considered statistically significant.

## Results

### Demographic and clinical characteristics

**Figure 3.**
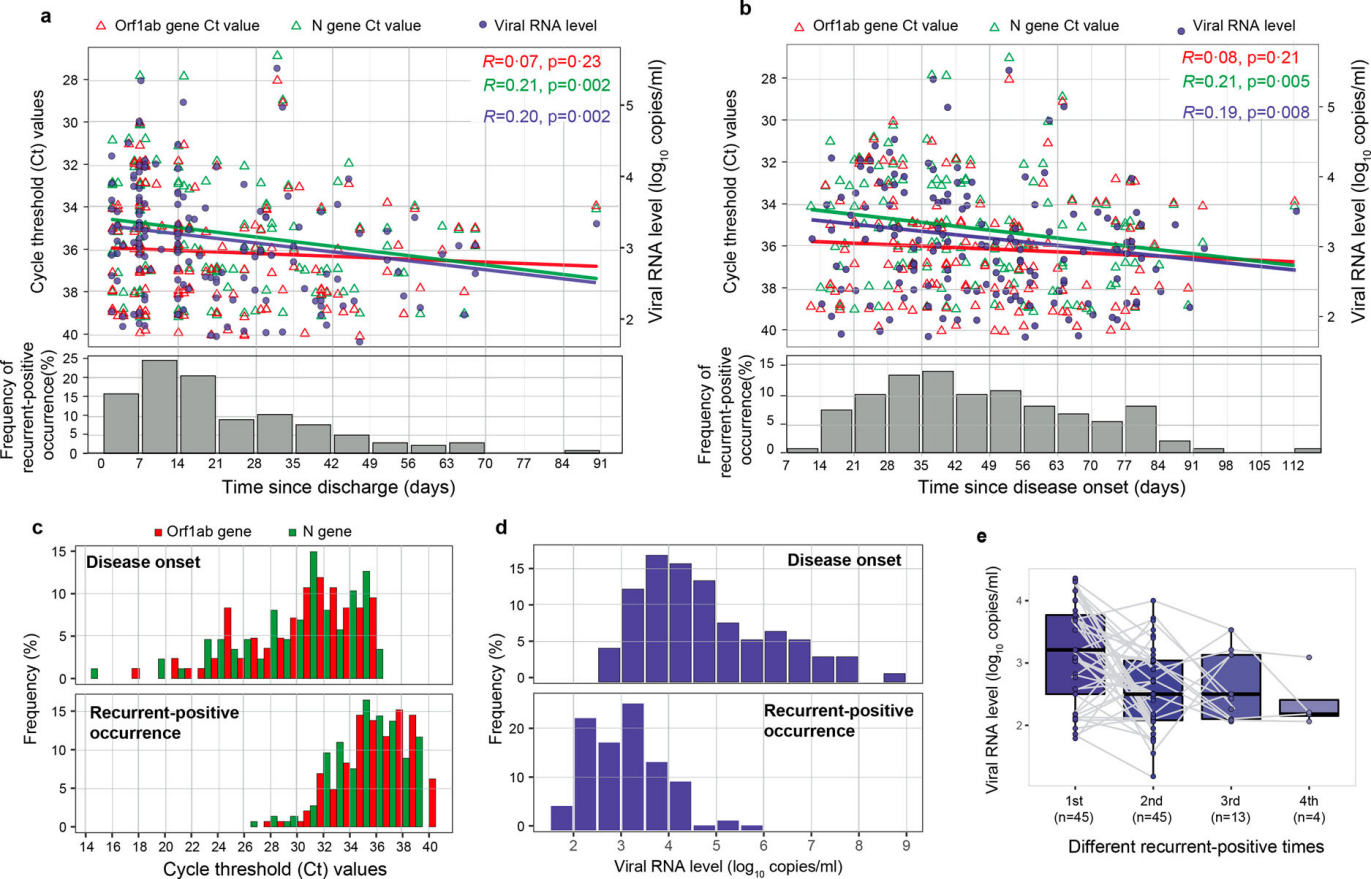
RT-qPCR cycle threshold (Ct) values and viral RNA levels in recurrent-positive patients. (a, b) Temporal distribution of Ct values (red and green triangles indicate the Orf1ab and N genes, respectively) and viral RNA levels (blue points) since discharge (a) or disease onset (b). The frequency of recurrent-positive occurrence is shown by grey bars. (c) Ct values of recurrent-positive patients at the time of disease onset (top) or recurrent-positive occurrence (bottom); colours indicate different target SARS-CoV-2 genes. (d) Estimated viral RNA level based on the correlation between viral RNA level and Ct value at the time of disease onset (top) or recurrent-positive occurrence (bottom). (e) Viral RNA level dynamics in multiple-recurrent-positive patients. Specimens from individual patients are linked by grey lines.

From February 1 to May 5, 2020, 504 recovered COVID-19 patients were discharged in Shenzhen. We excluded 25 of them from this study because of insufficient baseline information and enrolled the remaining 479 (438 symptomatic and 41 asymptomatic) patients (Figure 1). As of July 10, 93 (19%) recurrent-positive patients were identified, including 45 (9%) multiple-recurrent-positive patients with two (*n* = 32, 7%), three (*n* = 9, 2%), or four (*n* = 4, 1%) recurrent-positive results post-discharge (Figure 2). Of the 93 recurrent-positive patients, 70 (75%) were identified during their fourteen-day quarantine, and the remaining 23 (25%) were identified during follow-up. The median time from discharge to the first recurrent-positive was 8 days (95% confidence interval [CI]: 7–14 days; maximum: 90 days). The median times from discharge to final recurrent-positive and from disease onset to final recurrent-positive (viral RNA duration time) were 15 days (95% CI: 9–21 days; maximum: 90 days) and 46 days (95% CI: 38–53 days; maximum: 113 days), respectively (Table 1, Figure 2, and Figure 3a–b).

**Table 1.** Demographic and clinical characteristics of recurrent-positive and non-recurrent-positive patients.

	Recurrent-positive patients			Non-recurrent-positive patients (*n *= 386)	*p* value (recurrent-positive *vs* non-recurrent-positive)
	Total (*n* = 93)	Single recurrent-positive (*n *= 48)	Multiple recurrent-positive (*n *= 45)		
Age – median (95% CI)	34 (29–38)	31 (22–39)	38 (30–50)	45 (40–47)	<0.0001
Age – no./total no. (%)					
≤30 yr	38/93 (41%)	23/48 (48%)	15/45 (33%)	84/386 (22%)	0.0003
31–60 yr	46/93 (49%)	20/48 (42%)	26/45 (58%)	212/386 (55%)	0.41
≥61 yr	9/93 (10%)	5/48 (10%)	4/45 (9%)	90/386 (23%)	0.01
Sex – no./total no. (%)					
Female	57/93 (61%)	30/48 (62%)	27/45 (60%)	198/386 (51%)	0.11
Male	36/93 (39%)	18/48 (38%)	18/45 (40%)	188/386 (49%)	0.11
Hospitalization days – median, (95% CI)	20 (17–24)	18 (14–21)	24 (19–31)	21 (20–22)	0.84
Clinical severity on first admission – no./total no. (%)					
Asymptomatic	7/93 (8%)	4/48 (8%)	3/45 (7%)	34/386 (9%)	0.85
Mild	13/93 (14%)	6/48 (12%)	7/45 (16%)	42/386 (11%)	0.51
Moderate	69/93 (74%)	35/48 (73%)	34/45 (76%)	288/386 (75%)	1.00
Severe	3/93 (3%)	3/48 (6%)	0/45 (0%)	19/386 (5%)	0.67
Critical	1/93 (1%)	0/48 (0%)	1/45 (2%)	3/386 (1%)	1.00
Lymphocyte counts (10^9^/L)					
First admission – median (95% CI)	1.62 (1.45–1.78)	1.68 (1.42–1.93)	1.56 (1.33–1.86)	1.59 (1.45–1.83)	0.78
Discharge – median (95% CI)	1.70 (1.59–1.81)	1.70 (1.51–1.97)	1.68 (1.52–1.86)	1.82 (1.73–2.02)	0.07
C-reactive protein (mg/L)					
First admission – median (95% CI)	5.43 (4.00–8.60)	8.51 (2.82–20.44)	4.33 (3.00–6.07)	2.60 (1.20–4.94)	0.03
Discharge – median (95% CI)	1.74 (0.94–2.75)	2.15 (0.76–3.53)	1.66 (0.93–3.00)	1.68 (1.05–3.49)	0.74
Discharge to first recurrent-positive – median days (95% CI)	8 (7–14)	7 (7–14)	14 (8–14)		
Discharge to last recurrent-positive – median days (95% CI)	15 (9–21)	8 (7­–14)	35 (26–43)		
Onset to last recurrent-positive – median days (95% CI)	46 (38–53)	33 (29–40)	65 (54–75)		

There were more female (57/93, 61%) than male recurrent-positive patients (36/93, 39%, Table 1). This group was significantly younger (median age: 34 vs 45 years, *p *< 0.0001, Mann–Whitney U test) compared with the non-recurrent-positive patients, with 41% of recurrent-positive patients aged under 30 years vs 22% of non-recurrent-positive patients (*p *= 0.0003, Chi-square test). Recurrent-positive patients had a median hospitalization period of 20 days, and their clinical severity on first admission was mostly moderate (69/93, 74%) or mild (13/93, 14%). No recurrent-positive patients had underlying immunodeficiency diseases, and 14 recurrent-positive patients (15%) were treated with steroids (methylprednisolone and/or dexamethasone) during hospitalization. There were no significant differences between recurrent-positive and non-recurrent-positive patients in terms of hospitalization period, clinical severity on first admission, or steroid use (*p *> 0.05, Chi-square test). The C-reactive protein (CRP) level of recurrent-positive patients on first admission was significantly higher than that of non-recurrent-positive patients (*p *= 0.03, Mann–Whitney U test), but there was no significant difference in the CRP level on discharge (*p *= 0.74, Mann–Whitney U test). Compared with single-recurrent-positive patients, multiple-recurrent-positive patients had longer hospitalization periods (median: 24 vs 18 days, *p *= 0.02, Mann–Whitney U test) and viral RNA duration times (median time from onset to last recurrent-positive: 65 vs 33 days, *p *< 0.0001, Mann–Whitney U test), but had no significant differences in their other demographic or clinical characteristics.

During readmission, 67 of 93 recurrent-positive patients (72%) had no symptoms, while 26 (28%) had mild symptoms, including slight cough (18/93 [19%]) and chest tightness (3/93 [3%]). One patient (male, 12 years old) had a brief fever (temperature: 37.5 °C) for one day. Routine blood tests showed elevated interleukin 6 levels in one patient (male, 62 years old); all other patients had normal levels. Chest CT revealed that 18 (19%) patients had no pneumonia lesions and the lung lesions of the remaining 75 patients were improved (68/93, 73%) or unchanged (7/93, 8%) from first discharge. There were no significant clinical symptom differences between single- and multiple-recurrent-positive patients during readmission.

### Viral RNA level

Seventy-one (76%) recurrent-positive patients were identified by only positive nasopharyngeal swab results, 14 (15%) by only positive anal swab results, and 8 (9%) by positive results for both specimen types. All tested blood specimens (147/147) from recurrent-positive patients were SARS-CoV-2 RNA negative. The median Ct values of N and Orf1ab genes were 35 (95% CI: 35–36) and 36 (95% CI: 36–37), respectively, which are significantly higher than the corresponding values at disease onset (N gene median Ct: 31, 95% CI: 29–31; Orf1ab gene median Ct: 31, 95% CI: 30–32, *p *< 0.0001, Mann–Whitney U test; Figure 3c). Furthermore, recurrent-positive patient viral RNA levels ranged from 1.8 to 5.7 log10 copies/mL (median: 3.1, 95% CI: 3.0–3.2), which was significantly lower than the corresponding values at disease onset (median: 4.5 log10 copies/mL, 95% CI: 4.3–4.8, *p *< 0.0001, Mann–Whitney U test; Figure 3d), indicating low viral RNA levels in recurrent-positive patients. Most (89/93; 96%) recurrent-positive patients had a maximum viral RNA level of <5 log10 copies/mL. There was no significant difference in viral RNA levels between patients of different demographic and clinical categories, between single- and multiple-recurrent-positive patients, or between positive nasopharyngeal and anal swab specimens (*p *> 0.05, Mann–Whitney U test, Figure S2). There was a significant negative correlation between discharge time and viral RNA level (*R *= 0.20, *p *= 0.002, Spearman’s correlation test; Figure 3a), and the viral RNA level of multiple-recurrent-positive patients showed a declining trend as the number of recurrent-positive detections increased (Figure 3e).

### Antibody responses

To investigate the antibody responses of recurrent-positive and non-recurrent-positive patients, their SARS-CoV-2-specific surface spike protein receptor-binding domain (RBD) IgM, IgG, IgA, total antibody, and NAb were assessed. A total of 499 serum specimens were obtained from 78 recurrent-positive patients (289 specimens, 1–9 specimens/patient) and 94 non-recurrent-positive patients (210 specimens, 1–6 specimens/patient) within 14 weeks post-discharge (within 17 weeks post-disease onset). The IgM, IgG, IgA, total antibody, and NAb seropositivity rates at first post-discharge sampling (median: 24 days post-discharge) in recurrent-positive patients were 37% (29/78), 99% (77/78), 62% (48/78), 99% (77/78), and 88% (69/78), respectively, with a median NAb titer of 1:32 (95% CI: 1:16–1:32), which were not significantly different (*p *> 0.05, Chi-square test) from those of non-recurrent-positive patients (50% [47/94], 98% [92/94], 50% [47/94], 99% [93/94], and 92% [77/84], respectively; median NAb titer: 1:16, 95% CI: 1:16–1:32). For recurrent-positive patients whose specimens were collected on the day of recurrent-positive detection, these rates were 38% (18/48), 98% (47/48), 63% (30/48), 100% (48/48), and 91% (39/43), respectively, with a median NAb titer of 1:32 (95% CI: 1:16–1:32).

**Figure 4.**
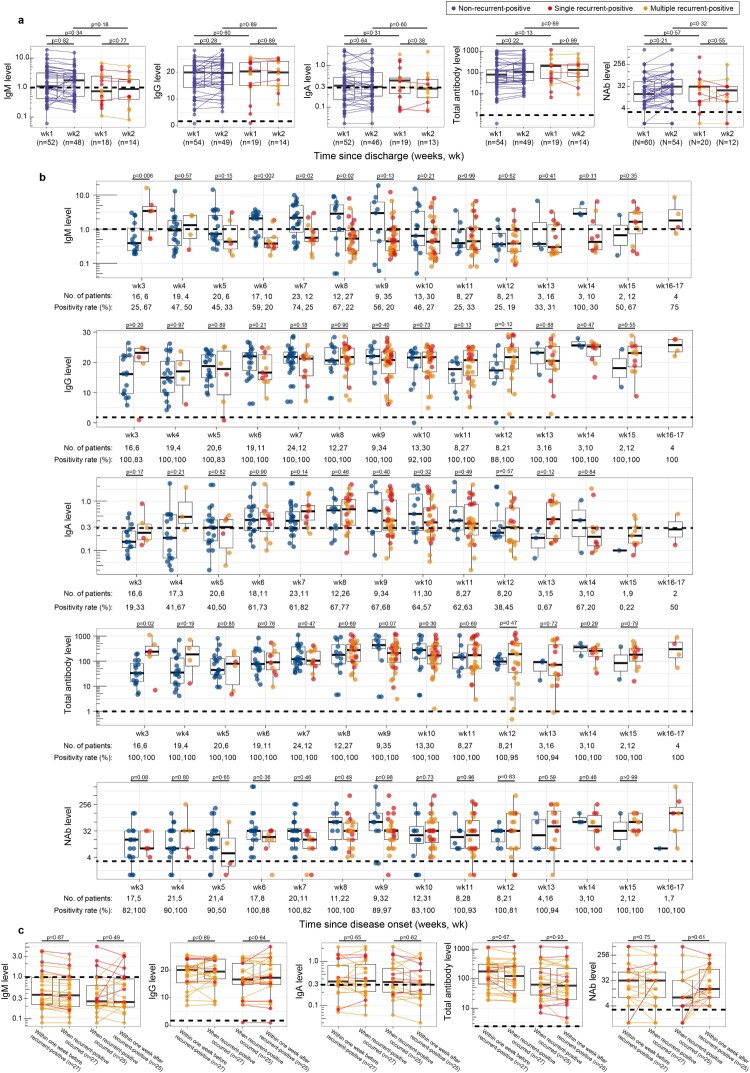
Serum SARS-CoV-2-specific antibody levels in recurrent-positive and non-recurrent-positive patients. (a–b) Levels of antibody against SARS-CoV-2 surface spike protein receptor-binding domain in recurrent-positive and non-recurrent-positive patients within two weeks post-discharge (a) or since disease onset (b). (c) Anti-SARS-CoV-2 surface spike protein receptor-binding domain antibody levels in recurrent-positive patients within one week before recurrent-positive detection, at the time of recurrent-positive detection, and within one week after recurrent-positive detection. Blue, red, and orange points show non-recurrent-positive, single-recurrent-positive, and multiple-recurrent-positive patients, respectively. Specimens from individual patients are linked by lines. Horizontal dotted lines indicate the positive detection threshold. The median value of multiple tests within one week was used to represent the antibody level of a patient.

We further quantitatively investigated the recurrent-positive and non-recurrent-positive patient antibody levels during different sampling periods. Seventy five percent of recurrent-positive patients were identified during their two-week quarantine post-discharge; no significant differences from non-recurrent-positive patients were identified in specimens from this period (Figure 4a). During our entire sampling period (3–17 weeks post-disease onset), no significant weekly differences were identified, except the IgM level in weeks 3 and 6–8, and total antibody level in week 3 (*p *< 0.05, Mann–Whitney U test, Figure 4b). Specifically, one (1%) and five (6%) recurrent-positive patients were negative for IgG and NAb, respectively, which is not significantly different (*p *> 0.05, Chi-square test) from non-recurrent-positive patients (IgG-negative: 3% [3/94]; NAb-negative: 8% [7/84]). Furthermore, we compared the recurrent-positive patient antibody levels on the day of recurrent-positive detection and within one week before and after recurrent-positive detection (when patients were viral RNA negative); no significant differences were identified (Figure 4c). Together, these results indicate that the SARS-CoV-2-specific anti-RBD antibody levels are generally similar in recurrent-positive and non-recurrent-positive patients, and in recurrent-positive patients regardless of current recurrent-positive detection. Additionally, there was a significant correlation between NAb titers and antibody levels (*R *> 0.40, *p *< 0.0001, Spearman’s correlation test), particularly for IgG (*R *= 0.73, *p *< 0.0001) and total antibody (*R *= 0.77, *p *< 0.0001), which indicates that they may be alternative indicators of NAb titer (Figure S3).

### Virus isolation and WGS

Virus isolation and WGS were performed to test whether live virus and/or complete viral genome, respectively, were detectable in recurrent-positive patients. Viral isolations of nine recurrent-positive patient nasopharyngeal specimens with representative Ct values (27–39, four specimens with a Ct value of <30 were included) were negative, as confirmed by testing the cell culture for SARS-CoV-2 RNA. WGS was successful for six of the nine specimens, but only genome fragments were obtained. The genome coverage of the specimens with the lowest Ct value (Ct: 27) was 55%, whereas the coverage of other specimens was <10%.

### Epidemiological investigations of close contacts

To assess whether recurrent-positive patients could spread the virus to close contacts, we conducted prompt epidemiological investigations of 23 recurrent-positive patients (identified during follow-up) on the day of recurrent-positive detection, which identified 96 close contacts. None showed clinical symptoms during the two-week follow-up, and all had negative SARS-CoV-2 RNA test results; 20 were tested for serum SARS-CoV-2-specific anti-RBD antibodies (IgM, IgG, and total antibody), and the results were also negative. Notably, one paediatric recurrent-positive patient was identified at 90 days post-discharge, after being in school for 11 days, and all 1,200 of his candidate contacts (teachers and classmates) showed no clinical symptoms during fourteen-day observation and had negative results from SARS-CoV-2 RNA tests. As of July 10, no close or candidate contacts of recurrent-positive patients had become confirmed COVID-19 patients. Additionally, a retrospective investigation of the contact history of 154 COVID-19 patients after February 1 found that none was epidemiologically related to our recurrent-positive patients. These results provide direct evidence that recurrent-positive patients have a low viral transmission risk.

### NAb titer in fully recovered patients

**Figure 5.**
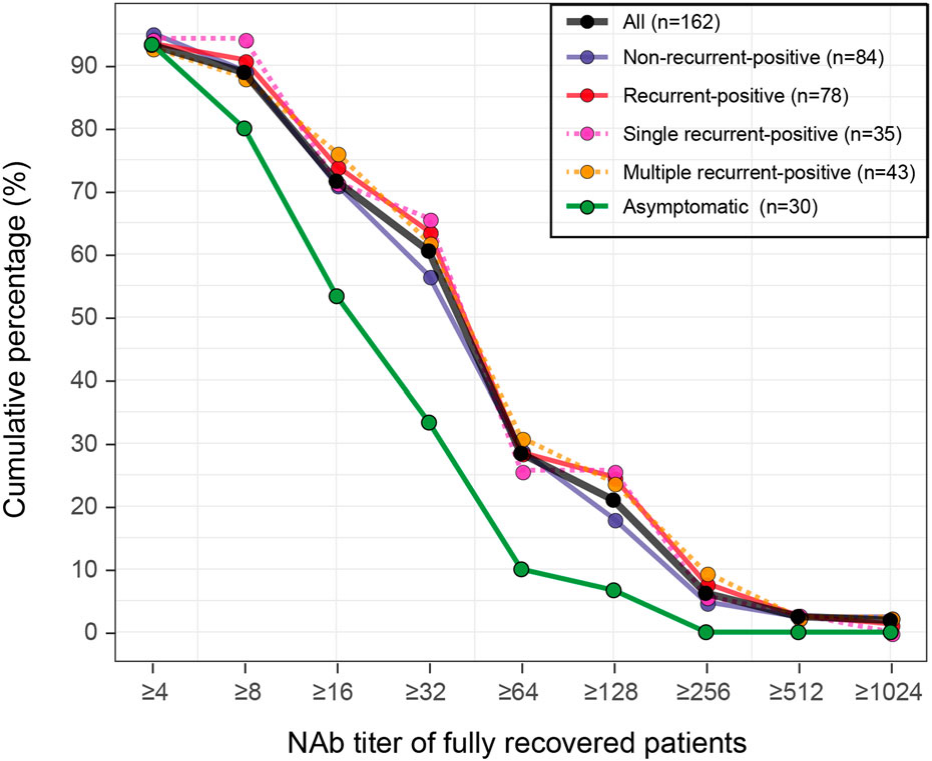
Reverse cumulative distribution curves of NAb titers in fully recovered patients. Colours show different types of patients.

All recurrent-positive patients were re-discharged after obtaining negative SARS-CoV-2 RNA detection results during quarantine. As of July 10, none of our recurrent-positive patients had any further recurrent-positive results from SARS-CoV-2 RNA tests, i.e. all were fully recovered (no longer/never recurrent-positive). Among the 479 fully recovered COVID-19 patients, NAb titers were tested in 162 (84 non-recurrent-positive and 78 recurrent-positive patients), 93% (151/162) of whom were NAb-positive with a median titer of 1:32 (ranged from 1:4 to 1:1024). Of the 30 asymptomatic patients, 93% (28/30) were NAb-positive with a median titer of 1:16, which was significantly lower (*p *= 0.0006, Mann–Whitney U test) than that of symptomatic patients. Notably, five patients’ NAb became positive during quarantine or follow-up, including three recurrent-positive and two non-recurrent-positive patients, whereas 11 fully recovered patients (six recurrent-positive and five non-recurrent-positive) remained NAb negative during our sampling period. Based on the reverse cumulative distribution curve principle [9], we analysed the NAb titer distribution at the end of quarantine for 162 fully recovered COVID-19 patients (Figure 5). Recurrent-positive and non-recurrent-positive patients had similar NAb titer distributions. Although some patients had a high NAb titer (28% with NAb titer of ≥1:64), 60% of fully recovered patients had NAb titers of ≥1:32.

## Discussion

To our knowledge, this is the first population-based study to comprehensively describe the viral RNA level and antibody response characteristics of recurrent-positive patients and evaluate their viral transmission risk. Recurrent-positive patients were characterized by younger age, mild or absent symptoms, and no disease progression. They generally had low viral RNA levels but long viral RNA durations (up to 113 days post-disease onset). Although the prolonged presence of SARS-CoV-2 RNA in COVID-19 patients has been reported [6,10], our results suggest that low levels of SARS-CoV-2 RNA persisted in some patients after both clinical recovery and initial viral-negative conversion. There are generally no significant differences in antibody or NAb levels between recurrent-positive and non-recurrent-positive patients, or in recurrent-positive patients over time (before, during, or after recurrent-positive detection), suggesting that recurrent-positive occurrence may not be related to humoral immunity. The low viral RNA levels and effective, long-lasting antibody responses in recurrent-positive patients, combined with the failed virus isolation, fragmented genome detection, and lack of close/candidate contact infections from these individuals, suggest that recurrent-positive patients pose a low risk of viral transmission.

By systematically monitoring SARS-CoV-2 RNA in recurrent-positive patients during quarantine and follow-up, we found that recurrent-positive patients accounted for 19% of recovered COVID-19 patients, which is close to most previous reports (15%–21%) [4,6] but much higher than one recent report where 3% (23/651) of recurrent-positive patients were identified in a routine health check of recovered patients [11]. Considering that multiple negative RNA tests were also identified in our recurrent-positive patients, differences in detected recurrent-positive patient proportions may be related to the viral RNA testing frequency. However, in the context of systematic follow-up and testing, recurrent-positive occurrence in recovered patients is unlikely to be rare.

Although recurrent-positive patients have been observed by multiple independent researchers [3–6] and government authorities, including the Korean CDC [12], the cause of recurrent-positive occurrence remains unclear, and several hypotheses have been proposed. 1) recurrent-positive might be due to false-negative SARS-CoV-2 RNA test results at discharge [6,13]. Here, in the 59% of recurrent-positive patients who had additional negative test results before their first recurrent-positive result, the sampling and testing were performed by the same technician using the same kits, minimizing the likelihood of false-negative results. 2) recurrent-positive could be due to post-discharge reinfection. Here, 75% of recurrent-positive patients were identified during quarantine, and those identified during follow-up did not report any contact with COVID-19 patients, making reinfection unlikely. 3) In people with low antibody levels or immunity, uneradicated virus could cause secondary infections [14]. We did not detect significant differences in antibody levels between recurrent-positive and non-recurrent-positive patients or in recurrent-positive patients over time, suggesting that humoral immunity may not be related to recurrent-positive occurrence. Additionally, none of the recurrent-positive patients had immunodeficiency diseases, and there was no significant difference in steroid treatment between recurrent-positive and non-recurrent-positive patients. However, more data are needed to verify the relationship between recurrent-positive occurrence and immunity, especially regarding cellular immunity. 4) recurrent-positive occurrence may be due to the shedding of “dead” virus particles. This possibility is consistent with our negative virus isolation results. However, failed viral isolation does not confirm a lack of live virus; Wölfel et al. [15] found that live virus cannot be successfully isolated when the viral load is below 6 log10 copies/mL. More sensitive live virus detection methods, such as identification of subgenomic messenger RNA [15], are needed to prove this hypothesis. Based on our data from SARS-CoV-2 RNA testing on 2,589 clinical samples collected from February 18 to May 5, eleven recurrent-positive patients were identified ≥30 days post-discharge (maximum: 90 days post-discharge), and all patients had recovered; therefore, we propose that recurrent-positive occurrence in recovered patients is due to their intermittent and non-stable excretion of low levels of viral RNA. However, further studies on the mechanism of recurrent-positive occurrence are needed.

Because SARS-CoV-2 RNA positivity does not necessarily translate to infectivity, we integrated multiple approaches to systematically evaluate the viral transmission risk posed by recurrent-positive patients. The viral RNA level can be a useful indicator for accessing transmission risk. Wölfel et al. [15] proposed that patients with a viral load of <5 log10 copies/mL posed a low transmission risk based on virus isolation results. Here, 96% of recurrent-positive patients had a maximum viral RNA level of <5 log10 copies/mL (range: 1.8–5.7 log10 copies/mL). Four recurrent-positive patients had a maximum viral RNA level of >5 log10 copies/mL, linked with a possible risk of viral transmission. To assess whether recurrent-positive patients shed live virus, we attempted virus isolation on the four specimens with a viral RNA level of >5 log10 copies/mL and five representative specimens with lower viral RNA levels. All nine specimens produced negative results. The low viral RNA levels and negative virus isolation in samples from the recurrent-positive patients indicate that their transmission risk is low.

WGS can be used to identify viruses with specific mutations, the presence of which may identify reinfection from another source. However, we obtained only genome fragments from the recurrent-positive patient specimens after SARS-CoV-2-specific amplification, including the specimen with the lowest Ct value (Ct: 27, viral RNA level: 5.7 log10 copies/mL), which limited our further investigation. In comparison, Lu et al. [16] found that sequencing reads can cover ≥90% of reference genomes with a Ct value of <30, irrespective of the amplification and sequencing approach. Although technique differences exist, the low genome coverage of recurrent-positive patient specimens suggests a low viral RNA level, further supporting the idea that recurrent-positive patients pose a low transmission risk.

The most effective way to assess the transmission risk of recurrent-positive patients is to conduct epidemiological investigations of their close contacts. When conducting epidemiological investigations on 790 close contacts of 285 recurrent-positive patients, the Korean CDC did not identify any infections [12]. However, the possibility of asymptomatic infections in those contacts was not excluded through SARS-CoV-2 RNA testing and antibody testing. Here, not only did all 96 close contacts and 1,200 candidate contacts show no clinical symptoms, they also had negative SARS-CoV-2 RNA test results, and 20 of them had negative antibody results, suggesting there were no asymptomatic infections. As of June 10, no COVID-19 cases have been reported among those contacts. These findings directly support our conclusion that recurrent-positive patients pose a low transmission risk. Furthermore, the recurrent-positive patients had high and long-lasting NAb levels, suggesting that they can effectively clear virus, which further reduces their viral transmission risk.

Whether COVID-19 convalescent patients are protected against future SARS-COV-2 infections is largely unknown [13,17]. NAb play important roles in virus clearance and are considered vital for protection against viral disease. Among the 162 fully recovered recurrent-positive or non-recurrent-positive patients who were tested for NAb, 93% (151/162) were NAb positive, with a median titer of 1:32, and their NAbs was maintained for up to 17 weeks post-disease onset, suggesting that most recovered patients obtained effective and long-lasting protection against future SARS-CoV-2 infection. Effective vaccines against SARS-CoV-2 infection are urgently needed to reduce the burden of COVID-19, and more than 120 candidate vaccines are currently being developing worldwide [18,19]. NAb titers in recovered COVID-19 patients can provide reference values for the vaccine humoral immunogenicity surrogate endpoints research in vaccine efficacy evaluations.

Our study has several limitations. First, this was a single-centre study conducted on all recurrent-positive patients from Shenzhen. Because there are differences in the discharge criteria and SARS-CoV-2 RNA testing methods among different cities and counties, our recurrent-positive incidence needs to be verified by multicentre studies. Second, we collected only nasopharyngeal swab, anal swab, blood and serum specimens based on current sampling policies; other specimen types, such as lower respiratory tract and sputum specimens, were not collected. Thus, the recurrent-positive incidence in this study represents a conservative estimation. Third, the systemic collection of serum specimens started mid-study, and serum specimens from recurrent-positive patients during their hospitalization were not available, which limited further investigations on the antibody level dynamics of recurrent-positive patients. Fourth, we only performed viral isolations and WGS on nine representative specimens from recurrent-positive patients; more samples were preferred to verify the presence/absence of live virus and clarify the viral genome characteristics of recurrent-positive patients. Finally, due to the strict management of recovered patients, most recovered patients were identified during quarantine and consequently had few close contacts. This study included the close contacts of only 23 recurrent-positive patients; larger scale epidemiologic studies are needed to further confirm the transmission risk posed by recurrent-positive patients.

In conclusion, our study found that intermittent detection of low levels of SARS-CoV-2 RNA in recovered COVID-19 patients is not rare and that the timing of recurrent-positive detection varies (up to 90 days post-discharge). Recurrent-positive may occur in recovered patients following intermittent and non-stable excretion of low viral RNA levels, rather than re-infection. The transmission risk posed by recurrent-positive patients is likely low. To better balance COVID-19 prevention and control with economic activities and to more effectively manage recovered patients, while minimizing the psychological impact on these individuals; we suggest that public health authorities can take a relatively relaxed approach to managing recovered COVID-19 patients. However, the follow-up and personal protection of recovered patients should be strengthened.

## Supplementary Material

EMI.Supplement-R1.docx
