# Supplementary material for: Viral RNA level, serum antibody responses, and transmission risk in recovered COVID-19 patients with recurrent positive SARS-CoV-2 RNA test results: a population-based observational cohort study
Source: Emerg Microbes Infect. 2020 Nov 5;9(1):2368–78. doi: 10.1080/22221751.2020.1837018 (PMC7655076; doi:10.1080/22221751.2020.1837018)

**Supplementary text**

**Methods**

**RT-qPCR and viral RNA level**

The total RNA of nasopharyngeal swab, anal swab, and serum specimens were extracted by using a High Pure Viral RNA kit (Roche). Quantitative reverse transcription PCR (RT-qPCR) tests targeting the N and Orf1ab genes of SARS-CoV-2 were performed using commercial kits (Zhongshan Daan Bio-Tech) on the day of sample collection. Duplicate ten-fold serial dilutions (5–5×10^7^ copies/mL) of standard plasmid DNA (Zhongshan Daan Bio-Tech) were prepared to generate a standard curve for analysis of the viral RNA level. Ct values were converted into viral RNA levels (copy/mL) based on the standard curve.

**Serum antibody assay**

The levels of IgM, IgG, and total antibody against the SARS-CoV-2 surface spike protein receptor binding domain in serum specimens were measured by using a chemiluminescence kit (Beijing Wantai Biotech) with a Caris200 automatic chemiluminescence instrument and are represented as cut-off indexes (COIs). The levels of IgA antibody against the SARS-CoV-2 surface spike protein receptor binding domain in serum specimens were tested by using indirect enzyme-linked immunosorbent assay (ELISA) kits (Beijing Hotgen Biotech). The optical density (OD) was read at 450/630 nm (OD_450/630_). The COI and OD_450/630_ are expressed as the average of two independent experiments. To define the cut-off for seropositivity, 169 and 128 serum specimens from confirmed patients and healthy persons were used as positive and negative controls, respectively. Specimens with COI values of >1 or OD_450/630_ values of >0.3 were considered positive.

**Virus neutralization assay**

Neutralization assays were performed using a SARS-CoV-2 virus isolate (20SF014/vero-E6/3, GISAID accession number EPI_ISL_403934) in biosafety level 3 (BLS-3) laboratories. Briefly, Vero-E6 cell lines were seeded in 96-well plates at a density of 1×10^4^–2×10^4^ cells in 100 µL of medium. After being heat-inactivated at 56 °C for 30 min, serum specimens were serially diluted as follows: 1:4, 1:16, 1:64, 1:256, and 1:1024. SARS-CoV-2 strain 20SF014/vero-E6/3 (titer: 104.67 TCID_50_/50 µL) was co-incubated with or without the serum for 2 h (37 °C, 5% CO_2_). A 100-µL aliquot of this mixture was added to the monolayer of pre-seeded Vero-E6 cells. SARS-CoV-2 virus preparations with titers of 100 TCID_50_/50 µL, 10 TCID_50_/50 µL, 1 TCID_50_/50 µL, and 0.1 TCID_50_/50 µL were used as positive controls. The plates were kept in an incubator set at 37 °C with 5% CO_2_. When the cells treated with the positive control virus displayed lesions, the cytopathic effect (CPE) of every sample was recorded. The highest titer of the serum at which half of the cells were protected and did not display CPE was considered to be the neutralizing antibody titer of the serum. The positive and negative controls used in the antibody assays were also used to define the cut-off for NAb seropositivity. Specimens with titers of ≥1:4 were considered as positive.

**Virus isolation**

Briefly, throat swabs specimens were filtered using a 0.22-µm needle filter, and then 100-µl samples were inoculated on Vero cells. After a 1-hour incubation at 37 °C with 5% CO_2_, the cells were washed with phosphate-buffered saline and transferred to cell culture flasks with medium for 72 h at 37 °C with 5% CO_2_. The cells were monitored for CPE with light microscopy daily for 10 days. When lesions were observed in 76%–100% of the cells, the flasks were placed in a freezer set at −70 °C and freeze-thawed 1–2 times before being harvested to improve the virus titer of the samples. Extraction of nucleic acids from harvested samples was performed with a High Pure Viral RNA Kit in accordance with the manufacturer’s instructions (Roche). Extracted RNA was tested for SARS-CoV-2 genes by using an RT-PCR assay (Zhongshan Daan Bio-Tech).

**Whole genome sequencing and analysis**

Reverse transcription was performed by using a SuperScript VILO cDNA Synthesis kit (Thermo Fisher). SARS-CoV-2-specific amplification library construction was performed by using an Ion Ampliseq Library Kit 2.0 (Thermo Fisher), and sequencing was conducted using an Ion Torrent S5Plus sequencer. Sequenced reads were aligned against a SARS-CoV-2 reference genome (BetaCoV/Wuhan/Wuhan-Hu-1/2019, NCBI accession number MN908947) with CLC Genomic Workbench V9.0 software with 90% identity to evaluate the genome coverage and identify mutations.

**Figure S1. Standard curve based on the RT-qPCR Ct value and viral RNA level of standard product.**


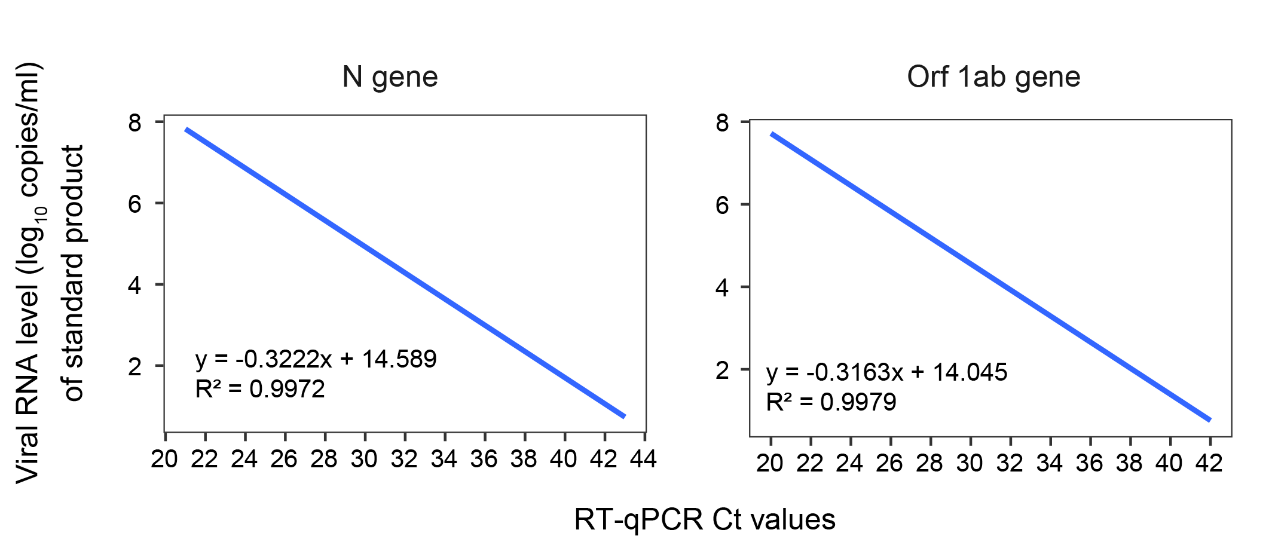


**Figure S2. Viral RNA levels in recurrent-positive patients of different demographic and clinical categories, single- and multiple-** **recurrent-positive patients, and different specimen types.**

**
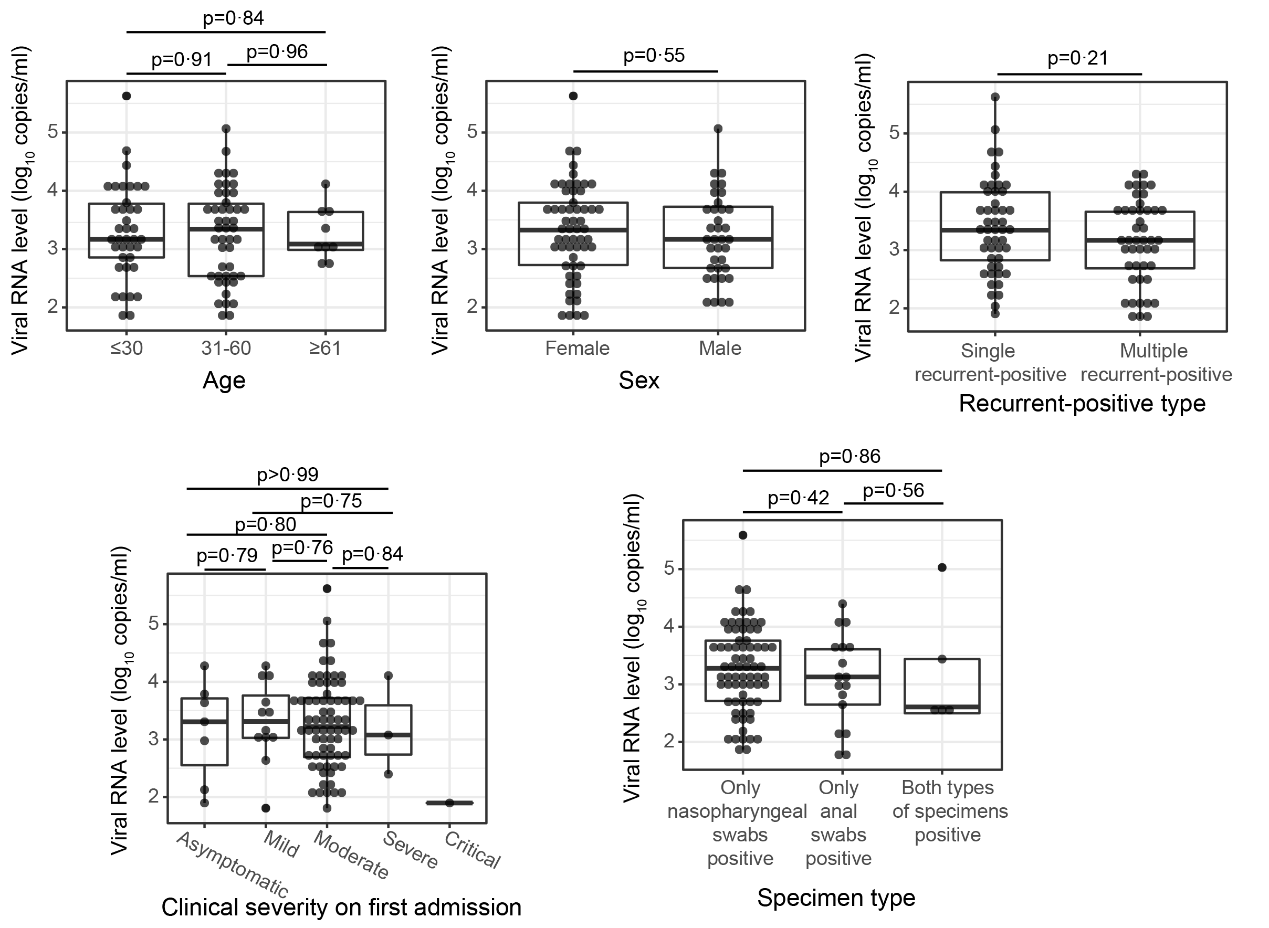
**

**Figure S3. Correlation between NAb titers and antibody levels.** Red and blue colours indicate recurrent-positive and non- recurrent-positive patients, respectively. Specimens with an NAb titer of <1:4 were assigned a value of 1.


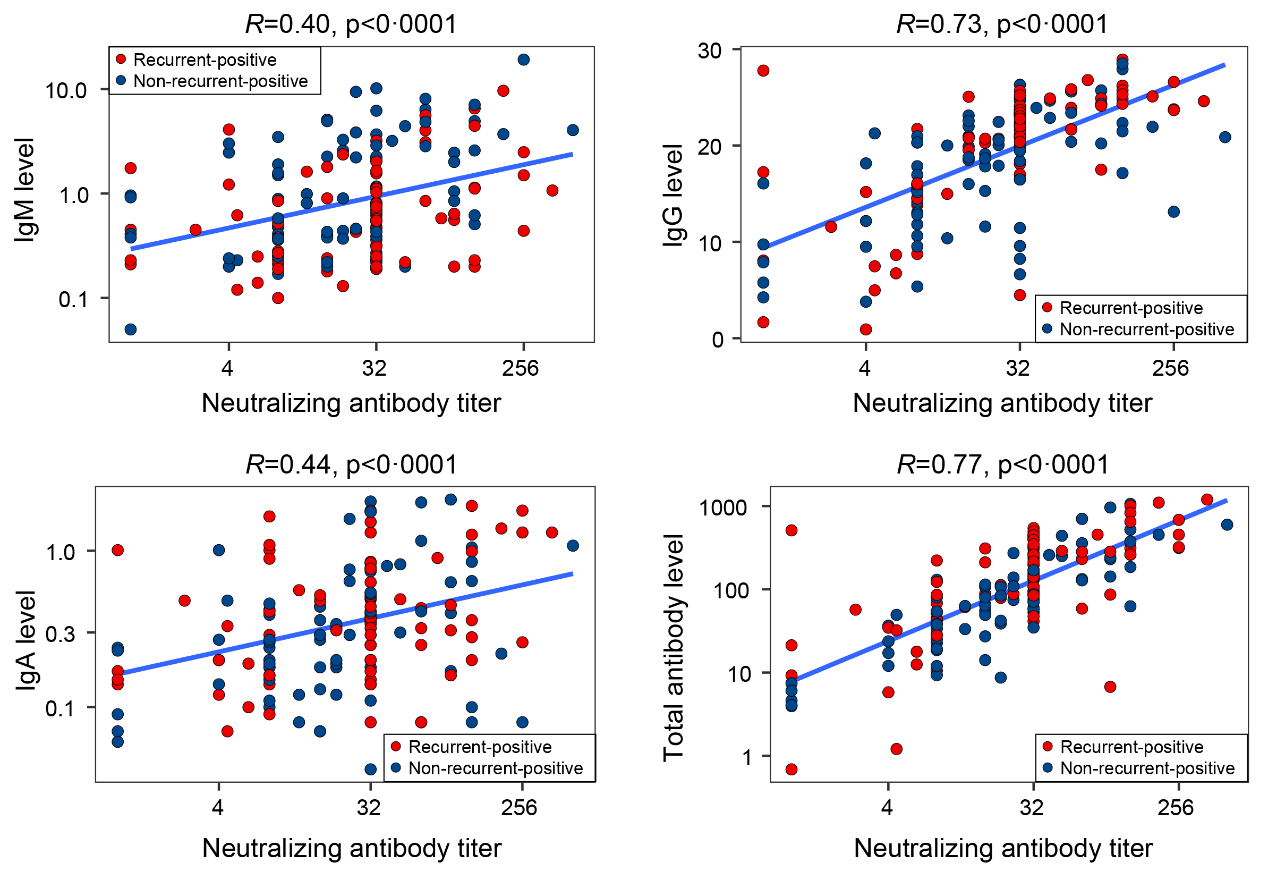

Supplement: EMI.Supplement-R1.docx [file TEMI_A_1837018_SM3700.docx]
